# Prenatal Exposures Are Associated With Worse Neurodevelopmental Outcomes in Infants With Neonatal Opioid Withdrawal Syndrome

**DOI:** 10.3389/fped.2020.00462

**Published:** 2020-08-27

**Authors:** Kristen L. Benninger, Teresa Borghese, Jason B. Kovalcik, Melissa Moore-Clingenpeel, Cherie Isler, Elizabeth M. Bonachea, Ann R. Stark, Stephen W. Patrick, Nathalie L. Maitre

**Affiliations:** ^1^Department of Pediatrics and Neonatology, Nationwide Children's Hospital, Columbus, OH, United States; ^2^Center for Perinatal Research, Abigail Wexner Research Institute, Nationwide Children's Hospital, Columbus, OH, United States; ^3^Biostatistics Core, Abigail Wexner Research Institute, Nationwide Children's Hospital, Columbus, OH, United States; ^4^Department of Neonatology, Beth Israel Deaconess Medical Center, Boston, MA, United States; ^5^Departments of Pediatrics and Health Policy, Vanderbilt University Medical Center, Nashville, TN, United States; ^6^Department of Hearing and Speech Sciences, Vanderbilt University Medical Center, Nashville, TN, United States

**Keywords:** *in utero* exposure, neonatal abstinence syndrome, neonatal opioid withdrawal syndrome, neurodevelopment, opioid

## Abstract

**Aim:** To define a developmental trajectory in infants with neonatal opioid withdrawal syndrome (NOWS) and determine whether the impacted developmental domain varies with the type of antenatal exposure.

**Methods:** We performed a retrospective cohort study of infants treated pharmacologically for NOWS and assessed using a standardized schedule for follow-up visits. We compared outcomes of the study population to published norms using one-sample *t*-tests. Multivariable models examined associations with exposures in addition to opioids.

**Results:** In our cohort of 285 infants with 9–12-months testing, 164 (55.7%) were seen at 3–4 months, and 125 (44%), at 15–18 months. The majority (58%) had intrauterine drug exposures in addition to opioids. Neurodevelopmental scores of infants with NOWS at 3–4 and 9–12 months were not different from published norms. Cognitive and language scores at 15–18 months were worse than published norms. Male sex, older maternal age, and additional barbiturate or alcohol exposure were associated with worse outcomes.

**Conclusion:** Infants with pharmacologically treated NOWS had development similar to unexposed infants during the 1st year but worse cognitive and language scores during the 2nd year. These data support the need for a prospective follow-up of large cohorts of infants with NOWS, with systematic assessments and an evaluation of contributing factors.

## Introduction

Neonatal opioid withdrawal syndrome (NOWS) is a significant public health problem in the United States, with ~1 baby born with opioid withdrawal every 15 min (1). Between 2006 and 2018 in Ohio, hospitals reported 17,373 discharges with a diagnosis of NOWS. During this period, the incidence of NOWS increased sevenfold (2).

Data from animal studies suggest a negative influence of opioids, stimulants and barbiturates on fetal brain development (3, 4). Opioid and other drug exposure during pregnancy can alter neonatal neurobehavioral and physiological responses to stimuli as measured by the NICU Network Neurobehavioral Scales (5, 6). Exposed infants display dysregulated behavior, high levels of stress/abstinence, and have difficulty modulating arousal, all of which can impair infants' interaction with caregivers and the environment (5–7). Neurobehavioral functioning among exposed infants typically improves during the 1st month of life, but stress and abstinence symptoms are still present in exposed infants at 6 weeks of age (5, 6). Further, some literature associates early neurobehavioral differences related to prenatal exposures with later developmental delays (7, 8). However, reports on the effect of intrauterine drug exposure on neurodevelopment in early human childhood are inconsistent (9–11). Several studies in infants and young children found that intrauterine opioid exposure was associated with cognitive and language impairments, while others found motor or visual impairments (10–13). In other reports, children with intrauterine opioid exposure demonstrated behavioral challenges at school age, particularly with deficits in attention and hyperactivity, executive function, and inhibitory control (14). More recent studies of children with NOWS found a higher rate of educational disabilities during early childhood (ages 3–8 years) (15) and poor academic performance from elementary through high school on standardized testing when compared to peers (15, 16). However, given the complex web of interconnected factors influencing development in this vulnerable population, the observed associations in these studies might be the result of numerous unmeasured confounding factors. Difficulties identifying consistent associations with outcomes after NOWS may be related to variations in dose, timing, and duration of exposure, polydrug use, and a changing profile of substances of abuse that may not be detected on routine toxicological screens (9, 17). In addition, children affected by NOWS frequently have socioeconomic risk factors associated with adverse neurodevelopment or additional exposures not detected on routine toxicology testing such as tobacco or alcohol, which makes it challenging to separate effects of intrauterine drug exposure from other risk factors (9, 17).

At Nationwide Children's Hospital, we routinely follow up infants who received pharmacologic treatment for NOWS in the Neonatal Intensive Care Unit's (NICU) Follow-Up clinic, using standardized assessments and neurological examinations at regular intervals in the first 2 years, similar to infants born preterm. We leveraged this systematic approach to conduct a retrospective cohort study to test the hypothesis that developmental impairment in infants with NOWS may not be evident in the 1st year of life, and the developmental domain that is impacted will vary with specific antenatal exposures.

## Materials and Methods

Data were collected from electronic medical records of the NICU Follow-Up Clinic at Nationwide Children's Hospital, Columbus, Ohio. We included all infants with a gestational age at birth ≥37 weeks who were pharmacologically treated for NOWS from 01/2011 to 12/2013 and who had a developmental assessment at 9–12 months of age. Infants with a diagnosis of NOWS who did not receive pharmacological treatment for withdrawal are not routinely seen in our NICU Follow-Up Clinic, and thus, neurodevelopmental testing was not available for these infants. We excluded infants if they had a genetic abnormality or major congenital malformation. The Institutional Review Board at Nationwide Children's Hospital approved this study.

In the NICU, intrauterine drug exposures were verified by toxicology testing of maternal urine, infant urine, meconium, or umbilical cord or by maternal report. Exposures were classified as opioids (methadone, morphine, heroin, fentanyl, oxycodone, and hydrocodone), benzodiazepines (BZD), barbiturates, marijuana (THC), or stimulants (amphetamines, methamphetamines, and cocaine). At the time of our study, pregnant women in our population were not treated with buprenorphine-containing products. Treatment protocols for pharmacologic management of NOWS at referring birth hospitals used modified Finnegan scores (18) to determine the need for pharmacologic treatment. However, these protocols were highly variable and included one or more medications (methadone, morphine, clonidine, and/or phenobarbital), with little inter-NICU consistency in initial dosing, escalation, addition of secondary medications or weaning. The only medication used for home weaning protocols across NICUs was phenobarbital, with weaning managed either by primary care providers or the NICU Follow-Up Program, with treatment duration ascertained from the medical record. Treatment dosing and duration in the hospital was unavailable for children referred from hospitals outside our network. If documented in the record, we also recorded information on maternal report of any other substances used during pregnancy that are not routinely tested on toxicology screening, such as alcohol or tobacco. We recorded insurance status (private vs. public) as a proxy for socioeconomic status.

### Follow-Up Schedule

The initial clinic visit typically occurred within 2–4 weeks of hospital discharge and included a physical exam, growth and nutritional assessment, social work needs assessment, and a medical evaluation for signs of withdrawal. Until 2013, infants with NOWS were next seen in the clinic at 9–12 months. After 12/2012, infants with NOWS were seen on the same schedule as extremely preterm infants. This included a developmental motor evaluation using the Test of Infant Motor Performance (TIMP) at 3–4 months, performed by trained physical and occupational therapists in the NICU Follow-Up Clinic (19, 20). The TIMP is a 42-item, normed assessment of postural and selective control of movement, necessary skills for functional motor performance (19, 20). It is validated from 32 weeks' gestational age to 4 months post-term age and involves observed and elicited motor assessment items (19, 20). The Bayley Scales of Infant and Toddler Development, Third Edition (Bayley III) is a widely-used, standardized assessment validated for identification of developmental concerns in children from the ages of 1–42 months (21). In our cohort, the Bayley III was used to assess cognitive, motor, and language development in children at 9–12 and 15–18 months of age. Assessments were performed in the NICU Follow-Up Clinic by certified examiners who are tested for reliability and adherence to standard administration as part of the Eunice Kennedy Shriver National Institute of Child Health and Human Development (NICHD) Neonatal Research Network (22). Examiners were not blinded to infant medical history. Scores derived included cognitive, motor, and language composite scores (standardized population mean 100, standard deviation 15).

### Analysis

All analyses were performed using SAS 9.3 (SAS Institute, Cary NC) and R (R: A language and environment for statistical computing. R Foundation for Statistical Computing, Vienna, Austria), with 2-sided *P* < 0.05 considered statistically significant. Comparisons of outcomes between the study population and published values for healthy infants were assessed using one-sample *t*-tests. All infants in the cohort were opioid-exposed, although many infants had additional drug exposures. Differences in outcomes between infants exposed only to opioids vs. those with additional exposures were tested using independent samples *t*-tests. Assumptions for all statistical tests (including approximate normality, equal variance, constant variance, and linearity) were verified using Shapiro-Wilk tests, qq-plots, and residual plots; a Satterthwaite correction for unequal group variances was applied where warranted. Effect sizes are reported using Cohen's *d* standardized mean differences. Differences between groups in the total number of antenatal drug exposures were assessed using Wilcoxon rank-sum tests. Comparisons on categorical variables were examined using chi-squared or Fisher's exact tests. To examine the association between the number of exposures and outcomes, Spearman correlation coefficients were used.

Multivariable linear regression was used to determine which antenatal exposures were independently associated with neurodevelopmental outcomes, after accounting for patient characteristics and treatment modality. Covariates were chosen based on variables that infer impact on neonatal and later outcomes in this and other patient populations (23–26). All models include race, sex, maternal age, private vs. public insurance, antenatal treatments, and prenatal exposures; all variables were retained in the model on clinical and theoretical grounds, regardless of statistical significance. Cohen's *F*^2^ values representing effect sizes are shown for key exposures and covariates.

Since research questions were not longitudinal in nature, all primary analyses were treated as if data were cross-sectional, using all available data for each visit. However, we performed two sensitivity analyses to confirm that our results were not substantially impacted by loss to follow-up: (1) we used repeated measures ANOVA to evaluate outcomes among patients with available data at both 9–12 and 15–18-months visits, and (2) we used linear mixed effects models using all available data across both visits, accounting for variable follow-up across patients (Supplemental Tables 1, 2).

## Results

Of the 449 NOWS new patient visits in our outpatient follow-up clinic during the study time period (1/2011–12/2013), 285 infants (63%) had 9–12-months developmental testing and were included in this study. Of these, 164 of 285 (55.7%) were born after 12/2012 and were also evaluated at 3–4-months visits. Almost half of the original cohort (125/285, 44%) was also seen for 15–18-months follow-up testing. The most common reason for loss to follow-up was invalid contact information.

All infants in our study cohort were prenatally exposed to opioids and pharmacologically treated for NOWS. Toxicology screening indicated that the majority (58%) of infants had exposures to 1 or more additional substances (Table 1). Concurrent exposure to stimulants (amphetamines, methamphetamines, and cocaine) was most common (45%), while exposure to barbiturates was least common (4%). A separate examination of each exposure (to opioids, BZD, THC, barbiturates, or stimulants) showed no differences by exposure status with respect to infant sex, insurance status, race, maternal age, or in total number of antenatal exposures.

**Table 1:** Study population characteristics (*n* = 285).

	***n***	**%**
**Sex (male)**	156	55
**Race**		
White	222	78
Black	18	6
Other	39	14
Unknown	6	2
**Medicaid**	271	95
**Drug exposure category**^**a**^		
Opioid	285	100
Benzodiazepine	30	11
Barbiturate	11	4
Marijuana (THC)	53	19
Stimulant(s)^b^	129	45
*Self-reported exposures*		
Alcohol	19	7
Tobacco	110	39
**Total number of documented drug exposure categories (excludes alcohol and tobacco)**		
1 (opioids only)	120	42
2 (opioids + 1 additional exposure category)	112	39
3 (opioids + 2 additional exposure categories)	48	17
4 (opioids + 3 additional exposure categories)	5	2

*This table lists characteristics of the study population. Race is self-declared*.

a *Total is >n for population due to multiple exposures*.

b *Stimulant drugs included amphetamines, methamphetamines, and cocaine*.

Infants with intrauterine exposure to any BZD, barbiturates, or stimulants were more likely to be treated with phenobarbital as an adjunct to opioids (70 vs. 38%, *P* = 0.0007, 82 vs. 39%, *P* = 0.005, and 51 vs. 33%, *P* = 0.002, respectively). In all but 19 infants, pharmacologic therapy for NOWS was completed before hospital discharge. Phenobarbital was the only medication used for continued treatment of NOWS in the outpatient setting. For those infants discharged home on phenobarbital, the average treatment duration after discharge was 91 days (range 19–233).

For the entire study population (infants with pharmacologically treated NOWS), we detected no differences between neurodevelopmental scores of infants with NOWS at 3–4 and 9–12 months compared to published norms for typically developing infants (Table 2) (19, 21). However, at 15–18 months, the study population had worse cognitive and language scores compared to published normal scores (Table 2) (21). Sensitivity analysis using longitudinal data in the 125 patients with both 9–12- and 15–18-months visits yielded similar results.

**Table 2:** Developmental scores: NOWS study cohort vs. published normal scores.

**Outcome**	**Study population**				**Published normal scores**^**a, b**^		***P***
	***N***	**Mean**	***SD***	**Range**	**Mean**	***SD***	
**3–4-months TIMP**	164	115.3	14.7	(57,141)	116	19	0.523
**9–12-months Bayley III**							
Cognitive	284	100.6	12.2	(15,125)	100	15	0.439
Language	285	100.4	12.8	(6,136)	100	15	0.598
Motor	285	99.7	13.5	(46,130)	100	15	0.704
**15–18-months Bayley III**							
Cognitive	125	96.9	12.5	(40,135)	100	15	0.007
Language	125	94.6	13.9	(50, 147)	100	15	<0.0001
Motor	125	99.1	11.2	(61,127)	100	15	0.359

a *Campbell et al. (19)*.

b *Bayley (21)*.

*Bayley III, Bayley Scales of Infant and Toddler Development, Third edition; NOWS, neonatal opioid withdrawal syndrome; SD, Standard Deviation; TIMP, Test of Infant Motor Performance*.

To investigate potential drivers of poor developmental outcomes in infants with NOWS, we analyzed associations with individual types of additional intrauterine drug exposure (Table 3). The total number of antenatal exposures was not associated with any adverse developmental outcomes at any time point. We also found no associations between individual exposures to BZD, THC, or stimulants and worse developmental scores. However, compared to infants not exposed to barbiturates, NOWS infants with antenatal barbiturate exposure had worse 15–18-months Bayley III language scores and worse 9–12 and 15–18-months Bayley III motor scores (Table 3). Scores were lower for all outcomes among infants exposed to barbiturates, with effect sizes ranging from moderately small (9–12-months Bayley III language scores, *d* = 0.43) to large (9–12- and 15–18-months Bayley III motor scores, *d* = 0.79 and 0.75, respectively). Maternal report of antenatal tobacco exposure was not associated with differences in outcomes. However, infants whose mothers reported alcohol use during pregnancy had significantly lower TIMP scores at 3–4 months (Table 3).

**Table 3:** Outcomes in NOWS infants with and without additional exposures.

**Outcome**	**No added exposure**			**Added exposure**			***d*^**a**^**	***P***
	***N***	**Mean**	***SD***	***N***	**Mean**	***SD***		
**BENZODIAZEPINE EXPOSURE**								
**3–4-months TIMP**	141	115.3	15	23	115	12.4	0.02	0.913
**9–12-months Bayley III**								
Cognitive	255	100.9	12.3	29	97.9	10.7	0.24	0.220
Language	255	100.4	12.6	30	100.6	14.4	0.02	0.928
Motor	255	99.7	13.5	30	99.7	14.1	0.00	0.987
**15–18-months Bayley III**								
Cognitive	113	96.4	12.6	12	101.7	10.5	0.42	0.167
Language	113	94.2	14.1	12	99.2	11.8	0.36	0.236
Motor	113	99	11.2	12	99.5	11.2	0.04	0.892
**BARBITURATE EXPOSURE**								
**3–4-months TIMP**	157	115.5	14.4	7	110.9	19.9	0.31	0.417
**9–12-months Bayley III**								
Cognitive	273	100.7	12.1	11	97.3	12.9	0.28	0.362
Language	274	100.6	12.8	11	95.1	13.4	0.43	0.161
Motor	274	100.1	13.4	11	89.6	13.7	0.79*	0.011*
**15–18-months Bayley III**								
Cognitive	119	97.1	12.6	6	92.5	9.4	0.37	0.378
Language	119	95	14	6	86.7	7.3	0.60*	0.151
Motor	119	99.5	11.1	6	91.2	10.6	0.75*	0.075
**MARIJUANA (THC) EXPOSURE**								
**3–4-months TIMP**	129	114.8	14.7	35	117.1	14.4	0.16	0.395
**9–12-months Bayley III**								
Cognitive	231	100.1	12.5	53	102.5	10.7	0.19	0.210
Language	232	100.6	11.7	53	99.7	17	0.07	0.642
Motor	232	99.2	14.1	53	101.8	10.4	0.19	0.137
**15–18-months Bayley III**								
Cognitive	103	96.2	11.4	22	100.2	16.7	0.32	0.171
Language	103	93	13.1	22	102.1	15.5	0.67*	0.005*
Motor	103	98.9	10.9	22	100.1	12.5	0.10	0.657
**STIMULANT**^**b**^ **EXPOSURE**								
**3–4-months TIMP**	83	115.9	14.6	81	114.7	14.8	0.08	0.598
**9–12-months Bayley III**								
Cognitive	156	99.1	12.6	128	102.4	11.5	0.27	0.023*
Language	156	99.4	13.3	129	101.6	12.2	0.17	0.154
Motor	156	98.9	13.5	129	100.7	13.5	0.13	0.271
**15–18-months Bayley III**								
Cognitive	69	97.3	12.1	56	96.5	13	0.06	0.741
Language	69	95	13.8	56	94.2	14.1	0.06	0.734
Motor	69	100.1	11.7	56	97.8	10.5	0.21	0.245
**ALCOHOL EXPOSURE**								
**3–4-months TIMP**	129	116.5	13.3	15	103.3	21.9	0.92*	0.037*
**9–12-months Bayley III**								
Cognitive	226	100.1	12.1	19	102.9	11.6	0.23	0.327
Language	227	100.1	13.5	19	102.7	7.2	0.20	0.397
Motor	227	99.8	13.4	19	98.7	12.1	0.09	0.722
**15–18-months Bayley III**								
Cognitive	107	96.2	12.7	15	101.3	9.5	0.41	0.136
Language	107	94.3	14.3	15	97.4	12.1	0.22	0.419
Motor	107	98.9	11.4	15	100.5	9	0.14	0.615
**TOBACCO EXPOSURE**								
**3–4-months TIMP**	74	115.3	14.8	70	115	15.1	0.02	0.896
**9–12-months Bayley III**								
Cognitive	135	99.2	12.8	110	101.6	11.1	0.20	0.128
Language	136	99.9	13.6	110	100.7	12.6	0.06	0.635
Motor	136	100	13.3	110	99.4	13.3	0.05	0.721
**15–18-months Bayley III**								
Cognitive	71	96.2	13.3	51	97.8	11.2	0.13	0.496
Language	71	95.2	14	51	93.9	14.2	0.09	0.607
Motor	71	100.3	10.5	51	97.5	11.8	0.25	0.174

a *Standard categories for Cohen's d effect sizes are as follows: small (0.2), medium (0.5), large (0.8)*.

Cohen's d values >0.5 and P < 0.05 are denoted with *.

b *Stimulant drugs included amphetamines, methamphetamines, and cocaine*.

*Bayley III, Bayley Scales of Infant and Toddler Development, Third edition; NOWS, neonatal opioid withdrawal syndrome; SD, standard deviation; TIMP, Test of Infant Motor Performance*.

Multivariable analysis showed no association between any NOWS pharmacologic treatment modality (methadone, morphine, phenobarbital, and/or clonidine) and any of the neurodevelopmental outcomes at the 3–4, 9–12, or 15–18-months time points. Of the patient demographic variables considered in our model, male sex and increasing maternal age were significantly associated with worse neurodevelopmental outcomes (Table 4). Male sex was significantly associated with lower TIMP (by 5.8 points), Bayley III cognitive scores at 9–12 months (by 5.5 points), Bayley III language scores at 9–12 months (by 4.6 points) and 15–18 months (by 5.0 points), and Bayley III motor scores at 9–12 months (by 4.1 points) and 15–18 months (by 4.8 points). Increasing maternal age was significantly associated with lower cognitive scores at 9–12 months (0.4 points lower per year older), lower cognitive scores at 15–18 months (0.6 points lower per year older), and lower motor scores at 15–18 months (0.5 points lower per year older). After controlling for treatment modality and patient demographics, patients exposed to alcohol had TIMP scores at 3–4 months about 10 points lower on average than those not exposed to alcohol (*P* = 0.03) (Table 4), and those exposed to barbiturates had Bayley III motor scores at 9–12 months about 11.9 points lower on average (*P* = 0.01) (Table 4).

**Table 4:** Multivariable analysis: 3–4–months TIMP, 9–12 and 15–18-months Bayley III scores in NOWS patients.

		**TIMP (motor)**							
		**Regression Estimate^**a**^ (SE)**			***F^**2**^*^**b**^**				***P***
**3–4 months**									
Male		−5.82 (2.67)			0.04				0.032*
Maternal age		0.00 (0.27)			0.00				0.992
Barbiturate exposure		−9.45 (6.95)			0.02				0.176
Stimulant exposure		−0.31 (3.02)			0.00				0.918
Alcohol exposure		−10.03 (4.43)			0.05				0.025*
	**Cognitive**			**Language**			**Motor**		
	**Regression Estimate^**a**^ (SE)**	***F^**2**^*^**b**^**	***P***	**Regression Estimate^**a**^ (SE)**	***F^**2**^*^**b**^**	***P***	**Regression Estimate^**a**^ (SE)**	***F^**2**^*^**b**^**	***P***
**9–12 months**									
Male	−5.50 (1.67)	0.06	0.001*	−4.56 (1.77)	0.04	0.011*	−4.07 (1.90)	0.03	0.034*
Maternal age	−0.39 (0.17)	0.03	0.027*	−0.09 (0.18)	0.001	0.611	−0.23 (0.20)	0.01	0.243
Barbiturate exposure	−1.91 (4.32)	0.001	0.659	−7.00 (4.58)	0.01	0.129	−11.92 (4.93)	0.03	0.017*
Stimulant^c^ exposure	3.39 (1.85)	0.02	0.068	2.25 (1.96)	0.01	0.253	1.67 (2.11)	0.003	0.430
Alcohol exposure	2.66 (3.02)	0.004	0.379	2.32 (3.20)	0.003	0.470	−0.48 (3.44)	0	0.890
**15–18 months**									
Male	−2.11 (2.59)	0.01	0.418	−5.04 (2.59)	0.04	0.055	−4.82 (2.07)	0.06	0.022*
Maternal age	−0.58 (0.28)	0.05	0.041*	−0.54 (0.28)	0.04	0.057	−0.46 (0.22)	0.05	0.044*
Barbiturate exposure	−5.84 (7.11)	0.01	0.414	−11.78 (7.11)	0.03	0.101	−9.09 (5.69)	0.03	0.113
Stimulant^c^ exposure	−3.87 (2.88)	0.02	0.182	−1.51 (2.87)	0.003	0.600	−4.14 (2.30)	0.04	0.075
Alcohol exposure	4.64 (4.06)	0.02	0.256	1.45 (4.05)	0.001	0.721	3.10 (3.24)	0.01	0.341

a *Adjusted for insurance type, race, NOWS treatment modality used (morphine, methadone, phenobarbital, and/or clonidine), benzodiazepine exposure, THC exposure, and tobacco exposure. Regression estimates are interpreted as a shift in mean outcome scores when the predictor is present, for all predictors except maternal age. The estimate for maternal age is interpreted as a score change for each 1-year increase in maternal age*.

b *Standard categories for Cohen's F^*2*^ effect sizes are as follows: small (0.02), medium (0.15), large (0.35)*.

Cohen's F^2^ values >0.15 and P < 0.05 are denoted with ^*^.

c *Stimulant drugs included amphetamines, methamphetamines, and cocaine*.

*Bayley III, Bayley Scales of Infant and Toddler Development, Third edition; NOWS, neonatal opioid withdrawal syndrome; SE, standard error; THC, marijuana; TIMP, Test of Infant Motor Performance*.

## Discussion

We report the neurodevelopmental outcomes of infants with pharmacologically treated NOWS seen on a schedule of standardized assessments similar to that used to follow up preterm infants. We found neurodevelopmental scores of infants with NOWS during the 1st year comparable to published norms, but that NOWS is associated with poorer cognitive and language scores at 15–18 months.

Our findings of neurodevelopmental scores comparable to published norms in infants with NOWS in the 1st year but poorer Bayley III cognitive and language scores at 15–18 months are not surprising. Higher-order developmental functions such as behavior, language, cognition, and executive function are not fully established and are difficult to evaluate prior to 18 months (27). Unfortunately, preschool and school-age neurodevelopmental testing was not conducted for our cohort. This limits our ability to comment on the longer-term clinical implications of the differences in cognitive and language scores at 15–18 months. Despite being the most widely used developmental assessment in high-risk infant follow-up, the predictive validity of the Bayley is inconsistent (28–30). The available reports on neurodevelopmental outcomes after antenatal opioid exposure and NOWS are also confounded by a variety of complex maternal and neonatal factors (11). However, consistent with our findings, published studies, including several recent meta-analyses report some degree of neurodevelopmental impairment, especially in preschool or school-age children, with impaired cognition, behavioral problems, impaired executive function, and academic challenges being most common (10–16, 31–33).

Most infants in our NOWS cohort were polysubstance exposed (58%). However, other than exposure to barbiturates or alcohol, we found no strong associations between worse developmental scores and either the number or type of exposures. Infants with intrauterine alcohol exposure had lower TIMP scores at 3–4 months, even after controlling for treatment modality and patient demographics. While this effect did not persist at 9–12- or 15–18-months testing, it is likely subject to underreporting and inconsistent documentation in the medical record given the well-known developmental consequences of prenatal alcohol exposure (34–36). Infants exposed to the combination of opioids and THC appeared to have slightly higher Bayley III scores at 9–12 and 15–18 months, compared to those exposed to opioids alone (Table 3). However, this finding disappeared upon further analysis (Table 4). We also found no associations between individual exposures to BZD, stimulants, or tobacco and developmental scores. Antenatal exposure to barbiturates in addition to opioids, although relatively rare (4%), was associated with worse outcomes, and these infants demonstrated developmental deficits at multiple evaluation time points. Use of phenobarbital as adjunct therapy for NOWS was not directly correlated with prenatal exposure to barbiturates. Antenatal barbiturate exposure itself might alter fetal brain development as seen in animal models that demonstrate neuronal apoptosis and alterations in cellular proliferation in the developing brain (37). In humans, intrauterine exposure to phenobarbital as compared to other antiepileptic treatments is frequently associated with worse long-term neurodevelopmental outcomes, specifically, cognitive impairment (37, 38). Alternatively, barbiturate use itself may be a marker of other factors that contribute to worse neurodevelopmental outcomes (i.e., social risk, maternal behavioral health status). Similarly, long outpatient medication weans, especially with phenobarbital as in our cohort, likely impact observed developmental outcomes (39). Our current NOWS treatment protocols aim to decrease the use of phenobarbital as an adjunctive therapy, using clonidine as an alternative adjunct and minimizing outpatient medication weans.

Male sex and increasing maternal age (each year older inferred a higher risk) remained risk factors for worse neurodevelopmental outcome after adjustment for exposures and NOWS treatment modality (Table 4). In other studies, male sex is independently associated with poorer neurodevelopmental outcome in infants born preterm, small for gestational age, and infants with hypoxic-ischemic encephalopathy (40). Males are more likely to demonstrate clinical signs of opioid withdrawal and are more likely to require pharmacologic treatment for NOWS (41, 42). The exact mechanism of this association has yet to be elucidated but has been attributed to differences in brain organization and genetic, epigenetic, and hormonal factors (42, 43). This biologic vulnerability of male fetuses to intrauterine adversity remains an important area of future research. While older maternal age is associated with more pregnancy-related morbidities, studies have found an inconsistent impact of maternal age on long-term neurodevelopment (44, 45). Worse neurodevelopmental outcome associated with older maternal age in our drug-exposed cohort may be an effect of greater cumulative maternal drug use with subsequent poorer health and nutrition, which are known to impact infant neurodevelopment (46).

Medication for opioid use disorder (MOUD) remains the standard of care for women with opioid-use disorder given its established maternal and neonatal benefits (10, 12). Unfortunately, we did not have data on whether mothers in our cohort participated in MOUD programs. It is likely that infants of these mothers were more represented in our cohort due to the support and education they receive during pregnancy and after birth. This may have biased our results toward more favorable outcomes overall than in a general population of opioid exposed infants whose mothers did not receive similar interventions. Future studies should continue to promote the important role of MOUD in pregnant women and its impact on improving outcomes. To allow accurate prediction, future studies of prospective cohorts should mitigate any bias that could result from loss to standard care follow-up by enrolling all infants regardless of likelihood of returning for follow-up, mother's participation in supportive programs, or attendance at all follow-up visits.

Our study has several limitations, many of which have been identified as common challenges in studies of antenatal opioid exposure and NOWS (9, 17). First, its retrospective nature only allows us to show associations and precludes attribution of causality. Second, half of our original cohort missed the evaluation at 15–18 months, potentially weakening estimates of severity or prevalence of delays. Loss to follow-up is common in studies of infants with NOWS, with developmental follow-up rates from published studies ranging from 40 to 90%. Smaller selective cohorts have the highest follow-up rates, while larger inclusive cohorts such as ours have lower rates. Similar to our study, most report retention of <70% of the original cohort beyond 12 months (10–13). Loss to follow-up contributes to the variability and inconsistency of outcome results. However, our finding of the association of poorer scores at this age is consistent with other published reports (10–13, 15). Improved prenatal care and MOUD programs with supportive rather than punitive approaches, clear and consistent communication throughout pregnancy and hospitalization and emphasis on parental involvement, mother-infant bonding, and education regarding the developmental trajectory are all approaches that can help improve participation and follow-up (47). The majority of our cohort was white (78%) and received public insurance (95%). This relatively homogenous demographic distribution is typical of the NOWS population in Ohio and throughout the country and may limit generalizability to other more heterogeneous settings (48–50). Other limitations include the lack of quantitative data on exposures (specific type, amount, frequency, and timing) and treatment, which limited our ability to study the impact of cumulative drug exposure. In addition, we relied on documentation in the medical record of alcohol and tobacco use during pregnancy, which in turn relied on maternal report; both exposures are likely under-reported and inconsistently documented in the medical record. Published literature reports that more than 90% of women with opioid use disorder smoke cigarettes during pregnancy, a significantly higher prevalence than the 40% in our cohort, confirming likely under-reporting and/or inconsistent documentation of this variable (51–53). We were also unable to evaluate the potential influence of genetic and epigenetic factors on development (54). Most importantly, we lacked socioeconomic data, information about maternal intelligence or education level, and environmental variables such as maternal nutrition and food insecurity, maternal mental health, consistency of prenatal care, trauma history, adverse childhood experiences, medical complications, and quality and stability of the child-rearing environment which all have a critical role in mediating long-term neurodevelopmental outcomes (9, 11, 17). Cognitive and language development are strongly influenced by early experiences, developmental stimulation, and stability of the social environment; the possibility remains that social environment may be more predictive of cognitive and language delays than prenatal exposures or neonatal NOWS treatment (9, 17, 26, 55–58). However, it is most likely that a complex web of interconnected factors all influence development in this vulnerable population (9, 17, 57) Information about maternal intelligence, home environment, and postnatal caregiver was unavailable in our cohort; collecting this key information in future studies is essential. Despite these limitations, our findings contribute to the growing knowledge of neurodevelopment in this high-risk population.

At this time, no published recommendations guide the follow-up of infants with NOWS to facilitate the screening and timely identification of developmental impairment and referral for appropriate intervention. Future studies may uncover specific exposures, or demographic or environmental factors that identify infants at highest risk of deficit. The most appropriate timing, instruments, and setting for follow-up should balance optimal neurodevelopmental surveillance with issues of accessibility, availability of specialized personnel, and health care costs. Our findings and those of others suggest an approach to developmental surveillance of infants with NOWS that could rely on the use of validated screening tools by the primary care provider during the 1st year, followed by full neurodevelopmental and behavioral testing using standardized, multi-domain assessments at later time points (i.e., 18–24 and 33–36 months) when higher-order functions, such as cognition, language, and behavior, emerge and can be more accurately tested (14, 27).

In conclusion, by applying validated assessments at standardized intervals designed for preterm infants, we found that infants with pharmacologically treated NOWS had development similar to un-exposed infants during the 1st year but worse cognitive and language outcomes during the 2nd year. These data may inform the development of large prospective and comprehensive follow-up studies, with a systematic testing of all children and accounting for other contributing factors including epigenetics and new protocols for management of infants with NOWS (54).

## Data Availability Statement

The raw data supporting the conclusions of this article will be made available by the authors, without undue reservation.

## Ethics Statement

The studies involving human participants were reviewed and approved by The Institutional Review Board at Nationwide Children's Hospital. Written informed consent from the participants' legal guardian/next of kin was not required to participate in this study in accordance with the national legislation and the institutional requirements.

## Author Contributions

KB, AS, and SP contributed to study interpretation, reviewed and revised all drafts of the manuscript, and approved the final manuscript as submitted. TB and JK designed the initial study, tested patients in the clinic, participated in data collection and entry, drafted the initial manuscript, and approved the final manuscript as submitted. MM-C designed, performed the final statistical analyses, and approved the final manuscript as submitted. CI participated in data collection and entry, tested patients in the clinic, drafted the initial manuscript, and approved the final manuscript as submitted. EB designed the initial study, participated in data collection and entry, drafted the initial manuscript, and approved the final manuscript as submitted. NM designed the study in its final form, carried out preliminary analyses, reviewed and revised all drafts the manuscript, and approved the final manuscript as submitted. All authors contributed to the article and approved the submitted version.

## Conflict of Interest

The authors declare that the research was conducted in the absence of any commercial or financial relationships that could be construed as a potential conflict of interest.
