# Supplementary material for: Prenatal Exposures Are Associated With Worse Neurodevelopmental Outcomes in Infants With Neonatal Opioid Withdrawal Syndrome
Source: Front Pediatr. 2020 Aug 27;8:462. doi: 10.3389/fped.2020.00462 (PMC7481438; doi:10.3389/fped.2020.00462)
Supplement: Supplementary file 1 [file Data_Sheet_1.docx]

Supplementary Material

| **Supplemental Table 1. Multivariable analysis: longitudinal data for NOWS patients with both 9-12 and 15-18 month Bayley III scores** | | | | | | |
| --- | --- | --- | --- | --- | --- | --- |
|  | **Cognitive** | | **Language** | | **Motor** | |
|  | Regression Estimate**^a^** (SE) | *P* | Regression Estimate**^a^** (SE) | *P* | Regression Estimate**^a^** (SE) | *P* |
| Male | -4.5 (1.5) | .003^*^ | -4.29 (1.63) | .009^*^ | -4.07 (1.6) | .011^*^ |
| Maternal Age | -0.43 (0.16) | .007^*^ | -0.16 (0.17) | .358 | -0.28 (0.17) | .098 |
| Barbiturate Exposure | -3.41 (2.40) | .253 | -9.2 (4.24) | .031^*^ | -10.07 (4.12) | .015^*^ |
| Stimulant^b^ Exposure | 1.29 (1.68) | .445 | 0.99 (1.80) | .5825 | 1.14 (1.74) | .513 |
| Alcohol Exposure | 3 (2.63) | .263 | 0.99 (2.86) | .73 | 1.19 (2.70) | .659 |
| ^a^Adjusted for insurance type, race, NOWS treatment modality used (morphine, methadone, phenobarbital, and/or clonidine), benzodiazepine exposure, THC exposure, and tobacco exposure. Regression estimates are interpreted as a shift in mean outcome scores when the predictor is present, for all predictors except maternal age. The estimate for maternal age is interpreted as a score change for each one-year increase in maternal age. | | | | | | |
|  |  |  |  |  |  |  |
| ^b^ Stimulant drugs included amphetamines, methamphetamines and cocaine  *p*-values <.05 are denoted with * | | | | | | |
| ***Bayley III,*** Bayley Scales of Infant and Toddler Development, Third edition; ***NOWS***, neonatal opioid withdrawal syndrome; ***SE,*** standard error; ***THC***, marijuana | | | | | | |

| **Supplemental Table 2. Multivariable Analysis: 9-12 and 15-18 month Bayley III scores in NOWS patients—All available data** | | | | | | |
| --- | --- | --- | --- | --- | --- | --- |
|  | **Cognitive** | | **Language** | | **Motor** | |
|  | Regression Estimate**^a^** (SE) | *P* | Regression Estimate**^a^** (SE) | *P* | Regression Estimate**^a^** (SE) | *P* |
| Male | -4.3 (1.5) | .006* | -4.59 (1.61) | .005* | -3.91 (1.66) | .020* |
| Maternal Age | -0.44 (0.16) | .006* | -0.28 (0.17) | .093 | -0.29 (0.17) | .094 |
| Barbiturate Exposure | -8.41 (3.8) | .029* | -10.56 (4.24) | .010* | -14.49 (4.15) | .001* |
| Stimulant^b^ Exposure | 1.25 (1.67) | .458 | 1.45 (1.77) | .413 | 0.68 (1.82) | .709 |
| Alcohol Exposure | 0.37 (2.56) | .885 | -0.38 (2.71) | .890 | -2.51 (2.84) | .369 |
| ^a^Adjusted for insurance type, race, NOWS treatment modality used (morphine, methadone, phenobarbital, and/or clonidine), benzodiazepine exposure, THC exposure, and tobacco exposure. Regression estimates are interpreted as a shift in mean outcome scores when the predictor is present, for all predictors except maternal age. The estimate for maternal age is interpreted as a score change for each one-year increase in maternal age. | | | | | | |
|  |  |  |  |  |  |  |
| ^b^ Stimulant drugs included amphetamines, methamphetamines and cocaine  *p*-values <.05 are denoted with * | | | | | | |
| ***Bayley III,*** Bayley Scales of Infant and Toddler Development, Third edition; ***NOWS***, neonatal opioid withdrawal syndrome; ***SE***, standard error; ***THC***, marijuana | | | | | | |
